# Enhancing Brain–Computer Interface Performance by Incorporating Brain-to-Brain Coupling

**DOI:** 10.34133/cbsystems.0116

**Published:** 2024-04-25

**Authors:** Tianyu Jia, Jingyao Sun, Ciarán McGeady, Linhong Ji, Chong Li

**Affiliations:** ^1^ Lab of Intelligent and Biomimetic Machinery, Department of Mechanical Engineering, Tsinghua University, Beijing, China.; ^2^ Department of Bioengineering, Imperial College London, London, UK.; ^3^ School of Clinical Medicine, Tsinghua University, Beijing, China.; ^4^ Beijing Tsinghua Changgung Hospital, Tsinghua University, Beijing, China.

## Abstract

Human cooperation relies on key features of social interaction in order to reach desirable outcomes. Similarly, human–robot interaction may benefit from integration with human–human interaction factors. In this paper, we aim to investigate brain-to-brain coupling during motor imagery (MI)-based brain–computer interface (BCI) training using eye-contact and hand-touch interaction. Twelve pairs of friends (experimental group) and 10 pairs of strangers (control group) were recruited for MI-based BCI tests concurrent with electroencephalography (EEG) hyperscanning. Event-related desynchronization (ERD) was estimated to measure cortical activation, and interbrain functional connectivity was assessed using multilevel statistical analysis. Furthermore, we compared BCI classification performance under different social interaction conditions. In the experimental group, greater ERD was found around the contralateral sensorimotor cortex under social interaction conditions compared with MI without any social interaction. Notably, EEG channels with decreased power were mainly distributed around the frontal, central, and occipital regions. A significant increase in interbrain coupling was also found under social interaction conditions. BCI decoding accuracies were significantly improved in the eye contact condition and eye and hand contact condition compared with the no-interaction condition. However, for the strangers’ group, no positive effects were observed in comparisons of cortical activations between interaction and no-interaction conditions. These findings indicate that social interaction can improve the neural synchronization between familiar partners with enhanced brain activations and brain-to-brain coupling. This study may provide a novel method for enhancing MI-based BCI performance in conjunction with neural synchronization between users.

## Introduction

A brain–computer interface (BCI) is a novel communication approach that links neural activities with external devices, bypassing the conventional neuromuscular pathway. BCI frees humans from the limits of our physical limbs by extending the number of degrees of freedom we have over our environment and offers a promising way toward movement augmentation [1], such as controlling a third arm for multitasking [2].

However, the large interindividual variability of BCI decoding accuracy keeps BCI still far from broad applications. Sufficient decoding accuracy not only ensures the stability and efficiency of the BCI system but also promises the effectiveness of its applications. In a clinical study of BCI, a 10 chronic stroke patients’ 12-week BCI intervention study revealed that the level of motor recovery was in positive correlation with users’ BCI decoding accuracy [3]. Decoding performance is mainly determined by the decoding algorithm and its inputs. Various pattern recognition algorithms have been proposed to adapt to diversified application scenarios, such as fingertip force intention recognition [4], limb’s different joint movements intention recognition [5,6], and self-paced movement intention recognition [7]. Apart from the decoding algorithm’s performance, inefficient BCI decoding accuracy is also partly due to differences in people’s ability to modulate cortical activities, which are the inputs of the decoding algorithm. It is reported that approximately 30% of stroke patients fail to achieve effective BCI control [8] and have been termed “BCI illiterate” [9]. Physiological features derived from cortical activities play a crucial role in BCI decoding. Therefore, increasing task-related neural activities during BCI control may serve as a novel approach for enhancing BCI performance.

Previous research has reported that the perception of multineural information from tactile [10], vestibular [11], and visual feedback [12] can greatly influence performance during BCI control. Tactile stimulation was introduced in a sensorimotor rhythm-based BCI, and results showed that brain activation and BCI performance were both enhanced due to tactile stimulation [10]. Previous studies proved that mirror neurons could be stimulated by action observing [13], which caused the activation of the corresponding brain regions [14]. Therefore, BCI was conducted in virtual reality, aiming to provide users with a more immersive visual experience, and results showed positive effects [12]. Additionally, somatosensory feedback was also introduced into a BCI paradigm, and results proved that somatosensory activities contributed to activation of the motor cortex, improving BCI performance [11]. The listed studies above explored the effects of sensory inputs on BCI performance and were performed in single-person BCI task scenarios. To this end, multisensory perception in the process of social interaction may also have a similar positive effect.

To explore social interaction between brains, hyperscanning was proposed to reveal features of brain-to-brain coupling [15]. Brain-to-brain coupling has been reported to be associated with better performance in many cooperation tasks [16]. One typical social interaction is handholding. Goldstein et al. [17] explored the effect of handholding on pain reduction and results showed that touch positively contributed to pain reduction. It was also reported that brain-to-brain coupling during touch contact correlated with the level of empathy [17]. Eye contact in social interaction has also been verified to modulate brain-to-brain coupling [18] and improve joint attention [19]. It was also reported that increased cross-brain coherence was only found during real eye contact compared with watching a face video [20], which highlights the irreplaceable value of live social interaction. BCI performance is closely related to human neural activities. The mixed sensory inputs during social interaction are assumed to make positive effects on humans’ neural activities and BCI-based downstream applications. For example, BCI-based rehabilitation training has been proven efficient in clinical applications. Considering patients’ affective state also plays an important role on clinical outcomes, the positive emotional effects evoked by human interaction potentially improve the clinical effects of BCI-based rehabilitation training. This provides a novel perspective in optimizing BCI-based human–machine affective interaction techniques in rehabilitation robots.

In this study, we explore brain-to-brain coupling during motor imagery (MI)-based BCI. Cortical activation and brain-to-brain functional connectivity were analyzed during 2 typical kinds of social interaction: eye contact and hand contact. Users’ BCI performance in different kinds of social interaction was also evaluated to reveal brain-to-brain coupling’s effects on BCI decoding accuracy.

## Materials and Methods

### Participants

A total of 12 pairs of friends (24 healthy participants, 10 males and 14 females) were recruited in this study as the experimental group. Each pair had been friends for at least 1 year. Moreover, 10 pairs of strangers (20 healthy participants, 10 males and 10 females) were recruited as the control group. Participants of each pair were of the same sex. No participant had experience with BCI or reported any mental or neurological diseases. All participants were informed of the experimental procedure before giving their written consent. This study was conducted according to the principles in the Declaration of Helsinki and approved by the Institutional Ethical Committee of Tsinghua University. This study was registered at the Chinese Clinical Trial Registry with the identifier: ChiCTR2300077585.

### Experimental design

**Fig. 1.**
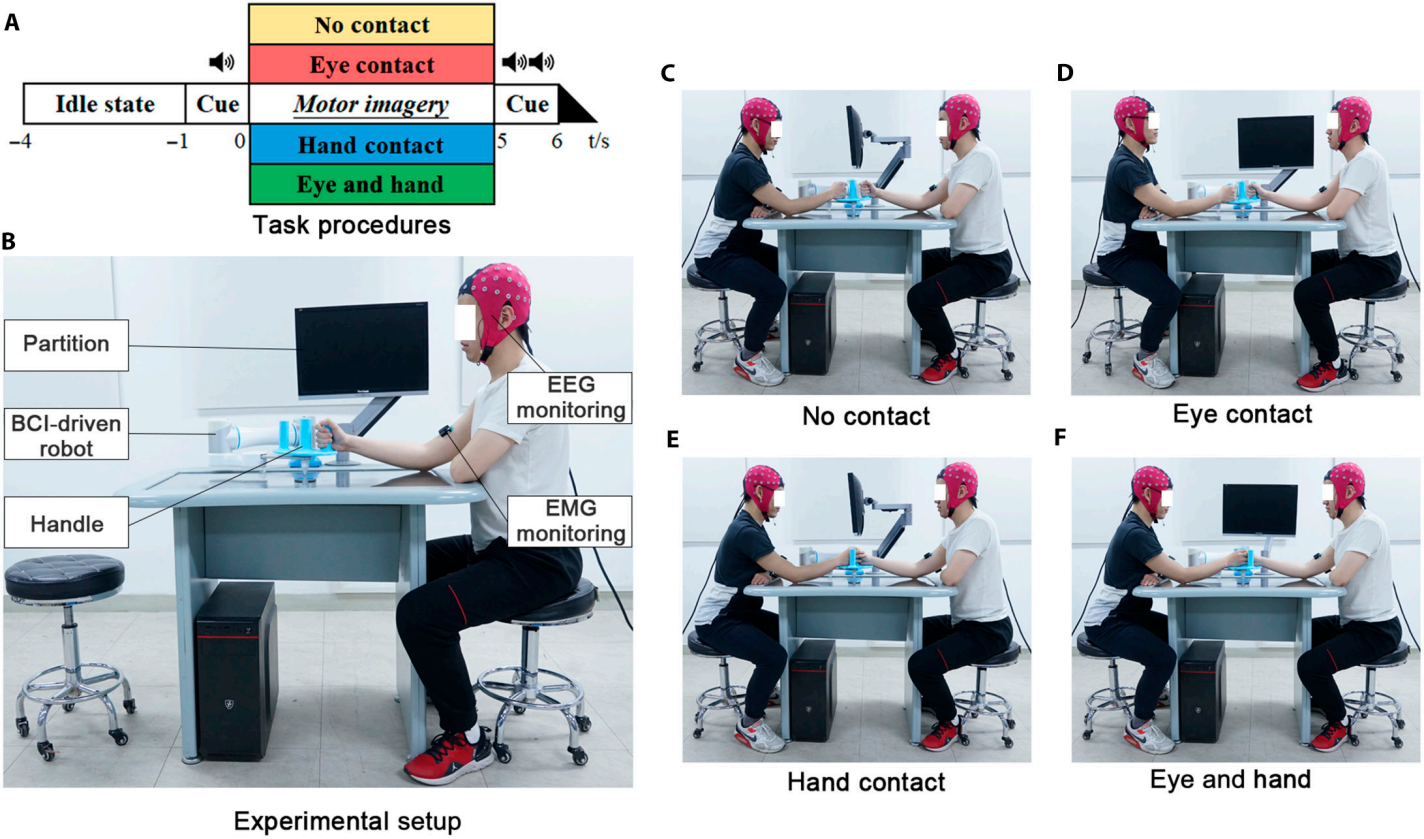
Experimental design. (A) Task procedures of 4 different experimental conditions. (B) Experimental setup. Motor imagery tasks under (C) no-contact condition, (D) eye contact condition, (E) hand contact condition, and (F) eye and hand contact condition. EMG, electromyography.

Upon participants’ arrival, the partners were asked not to communicate verbally until the end of the experiment. The participants were given no information regarding the effects of social interaction before and during the experiment. For each partner, they were randomly assigned the role of leader or follower. Partners were required to perform MI tasks under 4 different conditions: no contact, eye contact, hand contact, and eye and hand contact. Four sessions were corresponding to these 4 conditions that were arranged randomly for each pair of participants. Each session consisted of 60 trials, with the first 40 trials for BCI calibration and the last 20 trials for BCI decoding accuracy testing. The task procedure is shown in Fig. 1A. Each trial started with a 3-s idle state when the participants were required to rest. After that, an auditory cue indicated the start of MI. During MI, partners were required to imagine moving the robot from one preset side to the other side with their right hands. A stop auditory cue indicated the end of the task, where partners rested until the next start auditory cue.

The experimental setup is shown in Fig. 1B. There was a robot with a monitor (used as a partition) and a handle. The handle has 3 grips arranged on the left, right, and center of the handle. During the experiment, partners sat opposite each other on either side of the robot and performed the tasks. If no hand contact was required in the condition, partners were asked to use their right hand to hold the left grip and the right grip, respectively; if hand contact was required, the leader role should hold the middle grip and the follower role should hold the leader’s hand with their right hands. If no eye contact was required in the condition, the monitor was used as a partition and set between the partners; if eye contact was required, partners should keep eye contact during the whole experiment. Figure 1C to F shows 4 different conditions. During the experiment, participants were informed to avoid head or limb movements as far as possible. Surface electromyography from the biceps brachii and the triceps brachii was monitored to remind participants to avoid actual movement. There was a 5-min interval between each session for participants’ rest.

### Electroencephalography recording and preprocessing

Electroencephalography (EEG) was recorded using ANT eego™rt from 64 Ag/AgCl electrodes positioned according to the international 10-20 system. The sampling rate was 500 Hz, and the electrodes’ impedance was kept below 10 kΩ. EEG preprocessing was conducted in MATLAB 2020b with EEGlab toolbox [21]. EEG data was preprocessed based on the following steps: (a) common average reference with the exclusion of the EOG electrode; (b) band-pass filtered from 0.5 to 40 Hz using finite impulse response; (c) baseline corrections; (d) artifact removal using independent component analysis by an expert researcher; (e) cut into epochs following the trial markers.

### Comparisons of electrophysiological features

Power spectral densities were computed for each trial using the fast Fourier transform. The sensorimotor alpha band (8 to 13 Hz) has a strong association with upper-limb-related movement [22,23]. The average power within the alpha band across trials for each channel in each experimental condition was computed. In a simultaneous EEG–functional magnetic resonance imaging study [24], Zich et al. reported that a decrease in EEG sensorimotor rhythm amplitude correlated inversely with functional magnetic resonance imaging activation. Electrophysiological correlates of activated cortical areas involving in processing of motor-related behavior can be represented by the reduction of alpha band power, known as an event-related desynchronization (ERD) [10,12,25,26]. To compare cortical activity under the different experimental conditions, we analyzed the grand average temporal ERD, mean ERD during MI, and spatial distribution of mean ERD in the alpha band.

Time–frequency analysis was conducted using Morlet wavelets in the alpha band, with a step of 1 Hz. To maximize features and minimize noise, mean power spectral density (PSD) was computed across trials. To analyze ERD changes with respect to time, ERD was computed with the following equation:

$${\text{ERD}}_{c}(t)=\frac{{\mathrm{PSD}}_{c}(t)-{\mathrm{PSD}}_{c,\mathrm{ID}}}{{\mathrm{PSD}}_{c,\mathrm{ID}}}$$ (1)

where *c* is a specific channel, *t* is a specific time interval, and *PSD*_*c*,*ID*_ indicates the mean PSD during the idle state (from −3 to −1 s). The mean ERD across trials of each channel was computed with the following equation:

$$\mathrm{ERD}\_{\text{mean}}_{c}=\frac{{\mathrm{PSD}}_{c,\mathrm{MI}}-{\mathrm{PSD}}_{c,\mathrm{ID}}}{{\mathrm{PSD}}_{c,\mathrm{ID}}}$$ (2)

where *PSD*_*c*,*MI*_ indicates the mean PSD over a specific time interval of MI state (from 0 to 4 s). Right-hand movements have been proven to be correlated with the brain regions corresponding to the electrode position C3 [27]. Thus, EEG data from C3 was used to calculate the grand average temporal ERD and mean ERD.

Wilcoxon signed-rank test was used for comparisons of the average power and the grand average mean ERD (C3) between contact condition (eye contact condition, hand contact condition, or eye and hand contact condition) and no-contact condition, respectively. Post hoc analyses were performed using the false discovery rate (FDR) correction for 3 comparisons. Statistical significance was set to 0.05.

### Interbrain functional connectivity analysis

The Hilbert transform was used to estimate the instantaneous phases. The alpha band plays a role in synchronizing brain activity during nonverbal social interaction [28–30], and it is robust for measuring brain-to-brain coupling [28]. Likewise, human collaboration is also reflected by theta synchrony [31,32]. Therefore, alpha and theta bands were used to measure interbrain coupling. The Circular Correlation Coefficient (CCorr) [33] was calculated based on instantaneous phases of the interbrain electrode pairs. CCorr can reflect the covariation degree of the paired EEG signals and is less susceptible in detecting spurious hyperconnections [34]. CCorr between 2 signals *ϕ* and *φ* can be defined as follows:

$${\mathrm{CCorr}}_{\varphi ,\phi }=\frac{\sum_{k=1}^{N}\sin(\phi -\bar{\phi })\sin(\varphi -\bar{\varphi })\;}{\sqrt{\sum_{k=1}^{N}\sin^{2}(\phi -\bar{\phi })\sin^{2}(\varphi -\bar{\varphi })\;}}$$ (3)

Fisher’s Z transformation was used to normalize the CCorr to confirm its approximately normal distribution so that the subsequent statistical analysis could be carried out logically. Statistical analysis was based on the multilevel modeling approach to take into account the nested data structure and remove linear trends [17]. Here, we used the following statistical analysis algorithm with 3 steps to reduce the FDR. In the first step, a 2-way analysis of variance was used to test hypothesis H1 that the 3 contact conditions (eye contact condition, hand contact condition, eye and hand contact condition) showed higher brain-to-brain coupling level than the no-contact condition. Irrelevant interbrain electrode pairs with coincidental synchronization were removed in this step [34]. Based on the first step, only significant electrode combinations were analyzed in the second and third steps. In the second step, a paired-sample *t* test was used to test whether the eye contact condition or hand contact condition showed a higher brain-to-brain coupling level than the no-contact condition, aiming to reveal the influencing mechanism of the interpersonal eye contact and hand contact, corresponding to visual and tactile input. In the third step, a paired-sample *t* test was used to test whether the eye and hand contact condition shows a higher brain-to-brain coupling level than the other 3 conditions, which can reflect the different brain-to-brain coupling patterns related to the mixed influences of visual and tactile contact.

### Evaluation of BCI performance

During the experiment, the first 40 trials were used for BCI calibration and the last 20 trials for BCI testing. Due to a data recording failure in the experimental group, one follower’s test data in the eye and hand contact condition was missing. Thus, 23 participants were recruited in comparison of decoding accuracy between 3 experimental conditions (eye contact condition, hand contact condition, eye and hand contact condition) and no-contact condition, respectively. All 20 participants in the control group were recruited in comparison of decoding accuracy. EEG from all electrodes was used for motor intention decoding. Common spatial pattern and linear discriminant analysis were used for feature extraction and classification. A paired samples *t* test was used for the comparison of decoding accuracy with FDR correction for multiple comparisons.

## Results

### Motor imagery state power

**Fig. 2.**
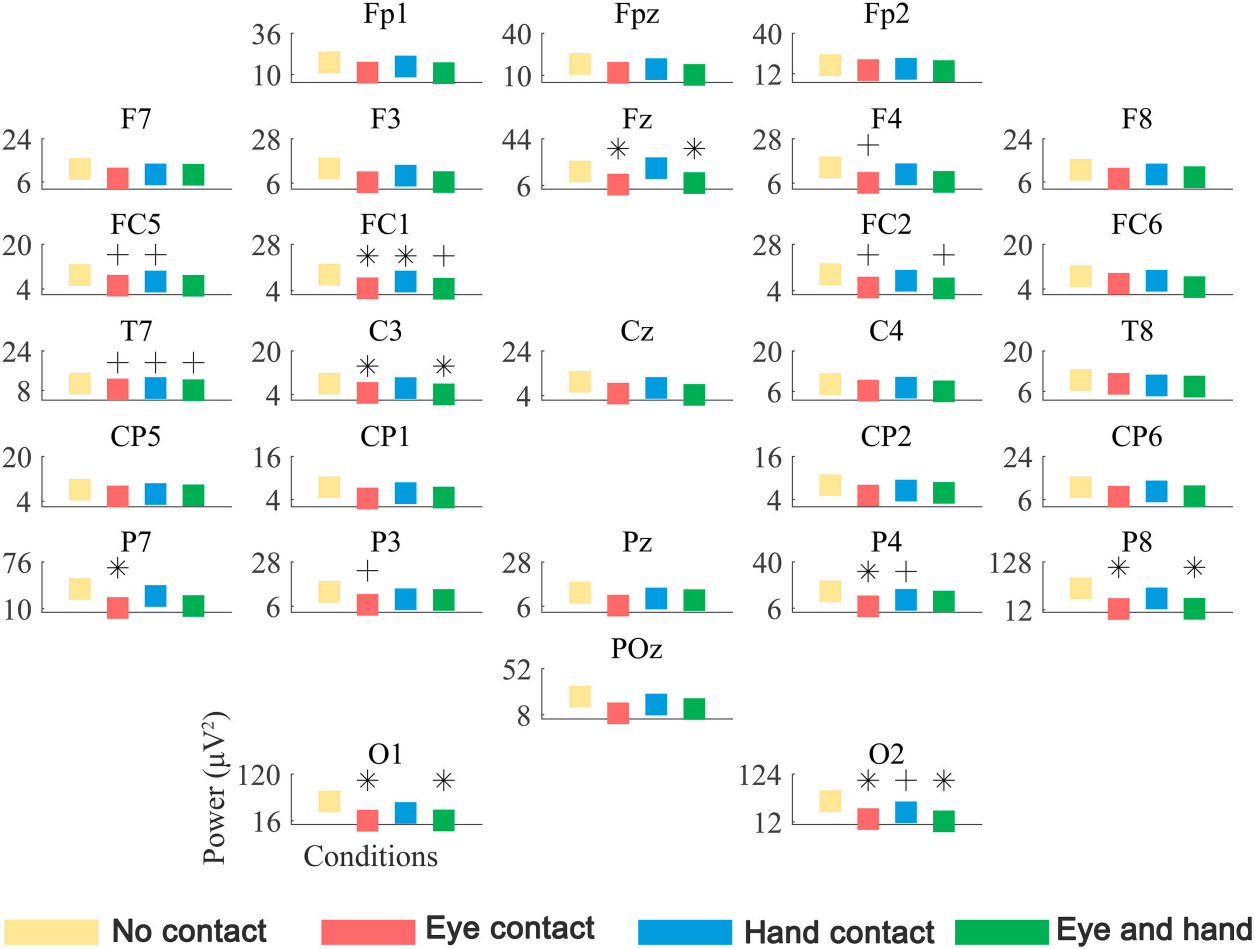
The power for each electrode over the 4 experimental conditions in the alpha frequency band of the experimental group. ∗ denotes a significant difference between the session with contact and the session without contact (*P* < 0.05); + denotes a difference that tended to be significant between the session with contact and the session without contact (*P* < 0.1).

Figure 2 shows the power for each electrode over the 4 experimental conditions within the alpha band of the experimental group. On the whole view, for each contact condition, alpha band power tended to be lower compared with no-contact condition. For the eye contact condition, significant decreases were observed for Fz, FC1, C3, P7, P4, P8, O1, and O2 (*P* < 0.05), and F4, FC5, FC2, T7, and P3 tended to be lower (*P* < 0.1). The above-listed channels are mainly distributed on frontal, frontocentral, central, parietal, and occipital regions. For the hand contact condition, there only existed a significant decrease for FC1 (*P* < 0.05) from the frontocentral region, and FC5, T7, P4, and O2 tended to be lower (*P* < 0.1). For the eye and hand contact condition, significant power-decrease channels were similar to those in the eye contact condition, consisting of Fz, C3, P8, O1, and O2 (*P* < 0.05). FC1, FC2, and T7 tended to be lower (*P* < 0.1). The above-listed channels are mainly distributed on the frontal, central, and occipital regions. Figure S1 (see Supplementary Materials) shows the power for each electrode over the 4 experimental conditions in the alpha band of the control group. Results showed that no significant changes were found on the bilateral sensorimotor cortex.

### Cortical activation

**Fig. 3.**
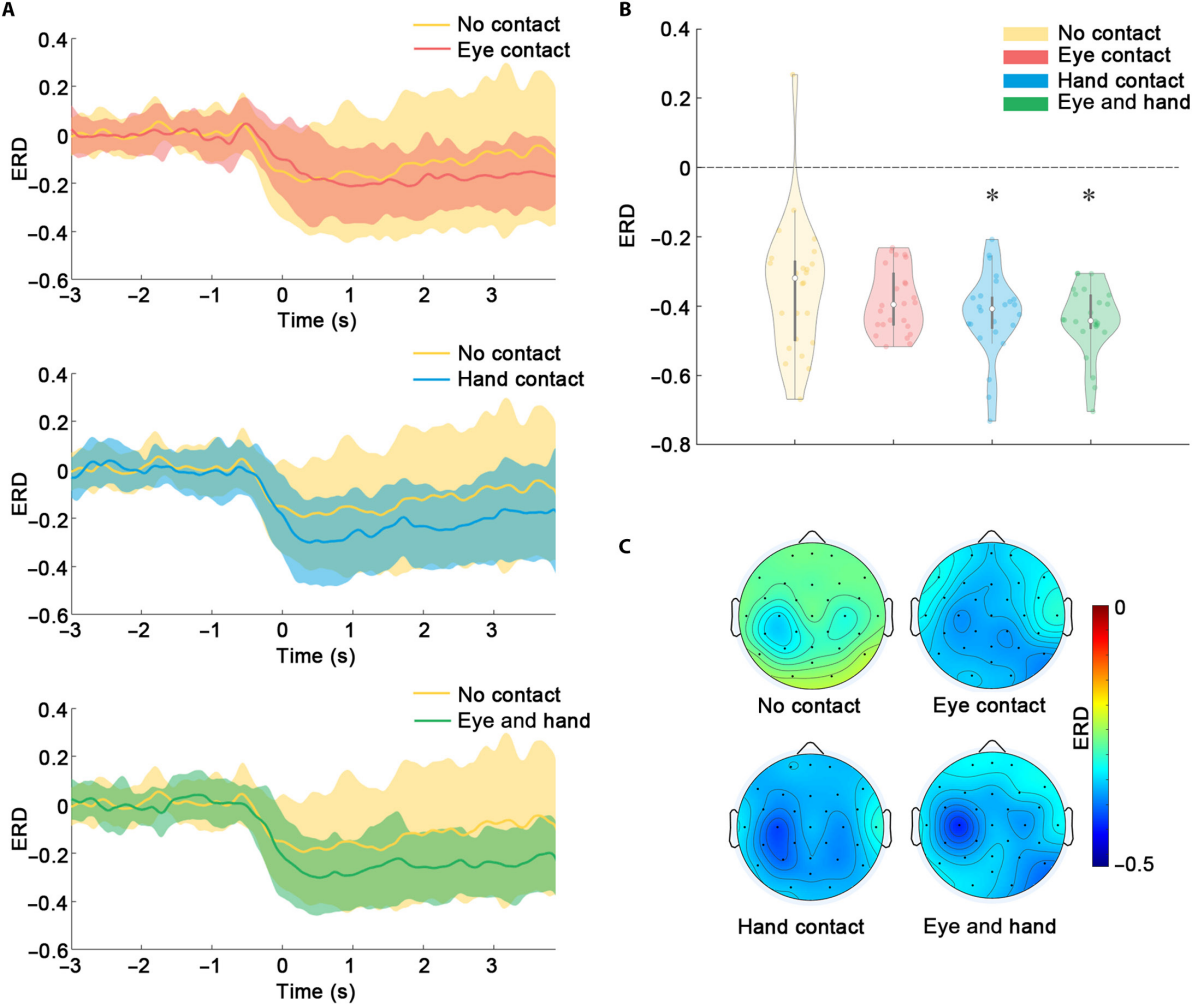
Comparisons of activation patterns between different experimental conditions in the experimental group. (A) The grand average temporal ERD (C3) under the 4 conditions. The shaded region represents standard deviation of temporal ERD across subjects and the solid line represents the average value. (B) The grand average mean ERD (C3) under the 4 conditions. (C) Spatial distribution of the grand average mean ERD under the 4 conditions.

To compare the cortical activation of participants in the experimental group under different interaction conditions, we analyzed the grand average temporal ERD (Fig. 3A), mean ERD (Fig. 3B), and spatial distribution of mean ERD (Fig. 3C). Figure 3A shows the temporal ERD of channel C3 under 4 different conditions. With hand and/or eye contact, greater temporal ERD was found compared with no contact (yellow lines). Figure 3B shows comparisons of mean ERD of channel C3. Greater mean ERD was observed in eye contact condition (−0.38 ± 0.09), hand contact condition (−0.42 ± 0.12), and eye and hand contact condition (−0.44 ± 0.10). Significant differences were found in hand contact (*P* < 0.05) and eye and hand contact (*P* < 0.05) compared with no contact. Grand average mean ERD topographic maps for 4 different experimental conditions are presented in Fig. 3C. MI tasks with 4 conditions mainly activated the contralateral sensorimotor cortex. Moreover, MI tasks with interaction showed greater activation than those with no interaction. These results implied that the social interaction enhanced cortical activation, especially the contralateral sensorimotor cortex. The opposite effect was found in the control group compared with the experimental group. Comparisons of the cortical activation of participants in the control group under different interaction conditions are shown in Figs. S2 and S3 (see Supplementary Materials). The results suggested that strangers’ average ERD under the no-contact condition tended to be greater than that under the contact condition.

### Interbrain coupling networks

**Fig. 4.**
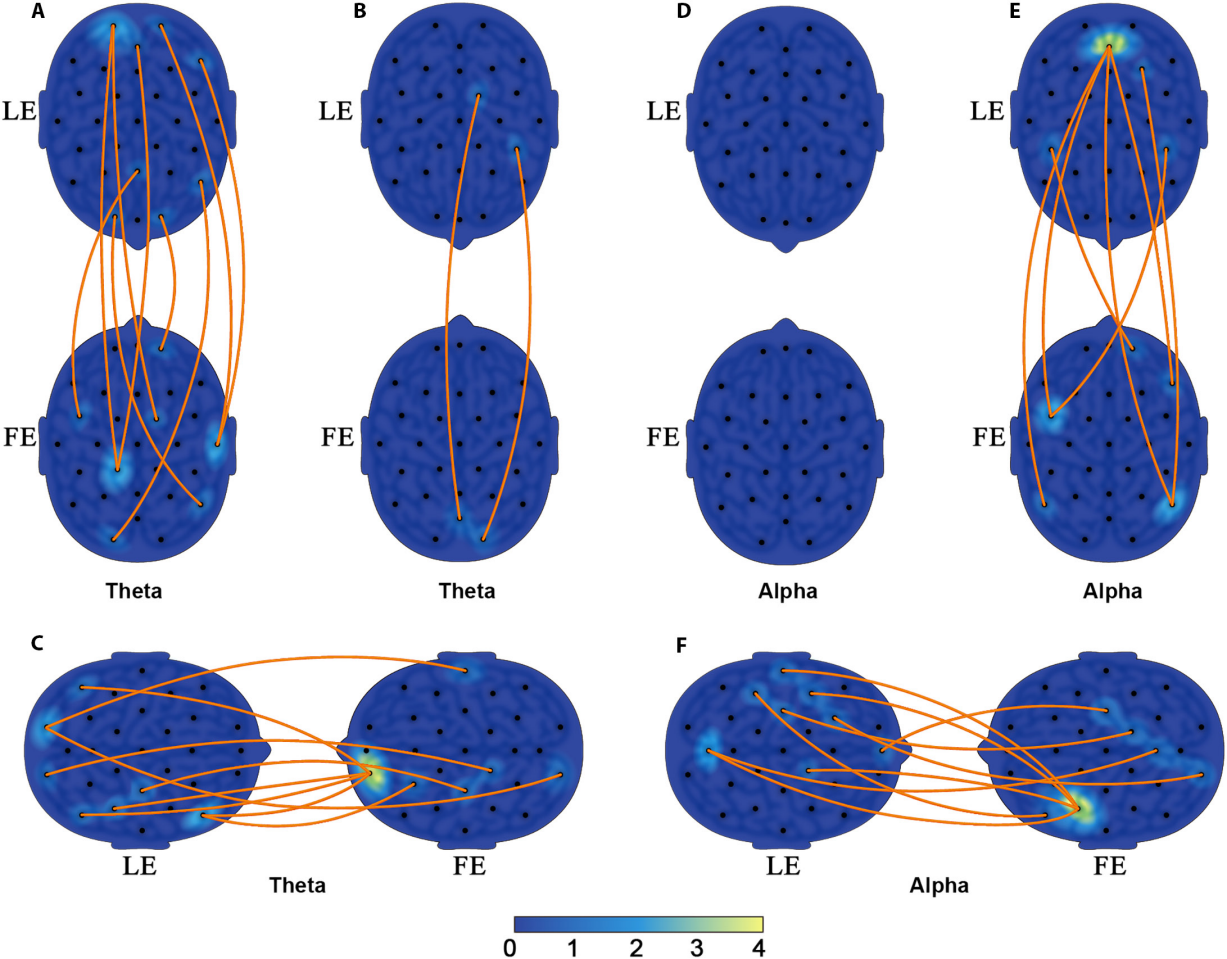
Interpartner EEG coupling between the leader (LE) and the follower (FE) in the experimental group. Theta band: (A) no-contact vs. eye contact conditions (9 links); (B) no-contact vs. hand contact conditions (2 links); (C) all other conditions vs. eye and hand contact condition (9 links); Alpha band: (D) no-contact vs. eye contact conditions (0 links); (E) no-contact vs. hand contact conditions (7 links); (F) all other conditions vs. eye and hand contact condition (9 links). The orange lines represent statistically significant coupling links between corresponding areas in the leaders’ and the followers’ brains. The head color reflects the number of links.

Interbrain coupling networks between the leader (LE) and the follower (FE) of the experimental group are shown in Fig. 4. Figure 4A and B and Fig. 4D and E depict the interbrain coupling networks for the second step of the analysis in the theta and alpha bands, respectively. In the theta band, eye contact condition formed an interbrain coupling pattern, which mainly involved the frontal, parietal, and occipital regions for leaders and the frontocentral and parietal regions for followers. In the alpha band, hand contact condition showed an interbrain coupling pattern between the parieto-occipital and frontocentral areas for leaders and the frontal and parietal areas for followers. However, there was nearly no increase in the hand contact condition of the theta band and the eye contact condition of the alpha band.

Figure 4C and F shows the interbrain coupling networks for the third step of the analysis, which compares the eye and hand contact condition with the other 3 conditions. The left head represents leaders, and the right head represents followers. In the theta band, interbrain coupling links were mostly between the right frontal, parietal, and occipital areas for leaders and left prefrontal regions for followers. In the alpha band, the eye and hand contact condition also demonstrated a large interbrain coupling pattern mostly between the parieto-occipital, left central regions for leaders, and left frontocentral regions for followers.

### Enhancement of BCI performance

**Fig. 5.**
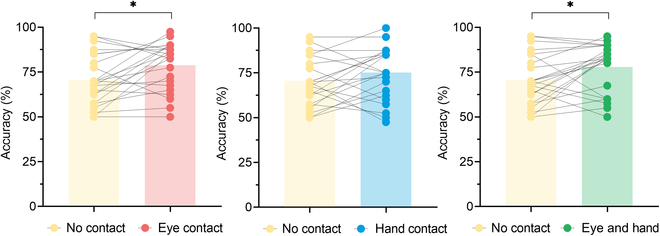
Comparisons of the experimental group’s BCI decoding accuracies in different experimental conditions. ∗ denotes a significant difference (*P* < 0.05).

Figure 5 shows comparisons of the experimental group’s BCI performance in different experimental conditions. Significant improvements of decoding accuracy were found in the eye contact condition (78.9 ± 13.4%) and in the eye and hand contact condition (77.9 ± 13.9%) compared with the no-contact condition (70.7 ± 14.2%) (*P* < 0.05), with no significant difference between no-contact condition (70.7 ± 14.2%) and hand contact condition (75.2 ± 15.1%) (*P* = 0.16). Comparisons of decoding accuracies in the control group under different interaction conditions are shown in Fig. S4 (see Supplementary Materials). A paired samples *t* test showed that neither eye contact (*P* = 0.06), nor hand contact (*P* = 0.07), nor eye and hand contact (*P* = 0.07) elicited a statistically significant change in BCI decoding accuracies in the control group.

## Discussion

In the present study, we investigated the effects of social interaction on neural oscillations and interbrain coupling during MI-based BCI training. We found that multimodal interaction can facilitate cortical activities, enhance brain activations, and promote interbrain coupling. This study may provide a novel method for enhancing cortical activation in conjunction with neural synchronization between partners.

Inspired by coordinated cooperation in solving complicated tasks, we introduced social interaction into BCI control. A familiar friendship may promise a good performance in social interaction. Therefore, in the experimental group, each pair of participants was required to be friends for at least 1 year. In addition, we recruited pairs of strangers to the control group for comparisons to explore whether social interaction is influenced by human familiarity. Interestingly, social interaction promoted cortical activation and contributed to better BCI performance in the friend-relationship group. However, no positive effects were found in the strangers’ group. This indicates that humans are fundamentally social and interpersonal relationship relies on their familiarity and closeness [35,36]. To further explore the different roles in social interaction, we set a leader and a follower in each partner to conduct BCI tasks. The leader–follower relationship is common in similar application scenarios. For example, in rehabilitation training, rehabilitation physicians assist patients to complete training tasks. Leader–follower cooperation may be the dominant mode of intelligent robot control in the future.

EEG power of alpha-band oscillations often represents excitability in the neural activity [37–39]. Therefore, alpha-band oscillations are regarded as an active activation of neural populations [40,41]. In the present study, the decreased power of the alpha band can imply attention improvement and global activation of brains [42]. Further, channels with alpha band power decrease mainly gathered in the left hemisphere, corresponding to the right-hand MI. In the eye contact condition, the increased involvement of frontal regions and frontocentral regions may represent the improvement of work memory performance [43,44], and involvement of central regions may represent the sensorimotor cortex activation [45]. Alpha band power decreases of parietal regions and occipital regions implied the specific involvement of visual spatial attention [46], which was consistent with the expected influence brought by eye contact. In the hand contact condition, more involved left frontocentral regions showed an improvement in attention during the MI. In the eye and hand contact condition, the involved cortical areas were similar to those in the eye contact condition, which indicates that eye contact plays a relatively more important role in neural activation as an interactive factor.

The MI task required participants to imagine right-hand movement, and the contralateral hemisphere, especially the contralateral sensorimotor cortex, was expected to be activated. Our ERD analysis further revealed that social interaction enhanced motor-related cortical activation. Previous studies showed that greater cortical activation measured by ERD could prove the efficiency of intervention methods in facilitating modulations of the sensorimotor cortex [10,12]. Besides, cortical and muscular activities are highly correlated with each other [47], and the cortical activation strength may indirectly reflect the enhancement of motor-related performance. Our study offers a novel method to facilitate motor-related cortical oscillations through social interaction. Moreover, ERD may be absent or reduced for stroke patients with brain deficits. The enhancement of ERD has been proven beneficial for recovery and also serves as an indication of rehabilitation [48]. A recent clinical trial with 770 patients revealed that robot-assisted training did not display superiority compared with usual care for motor recovery [49]. We may infer that, during the usual care, interactions between the patients and rehabilitation physicians play a crucial role in facilitating recovery that is absent from the robot-based training. Adding social interaction in robot-based rehabilitation training may further promote recovery efficiency.

It has been commonly reported that theta and alpha bands play an important role in neural coupling [16,50–52]. Therefore, interbrain coupling networks within theta and alpha bands were analyzed in this study. In past studies, the theta band was bound up with work memory, cognitive control, and behavior adjustment [53], which are necessary for social interaction [54]. Thus, in the theta band analysis of the eye contact condition, the increased involvement of frontal and parietal areas may indicate the improvement of work memory and cognitive control [55,56]. Apart from that, it can be regarded as activation of the visual cortex for the increased involvement of occipital areas, which were directly associated with mutual eye contact. Moreover, different from the eye contact condition, in the alpha band analysis of the hand contact condition, interbrain networks were asymmetrical between leaders and followers, which can be explained by the differential roles during the MI tasks. For leaders, clustered networks in parieto-occipital areas implied more rapid and effective decision-making during interbrain synchrony and cooperation [32]. For followers, mainly areas of the right frontal and left frontocentral regions and temporoparietal area were involved, which may be associated with the improvement of behavior coordination and attention orientation during the tasks [57–59]. At the same time, in the eye contact condition of the alpha band, no increased change was observed. However, power decreases of the alpha band were extremely significant, compared with poor functional connectivity in the eye contact condition, which can be a symbol of distraction to some extent. As shown in Fig. 4C and F, for the third step of the analysis in the theta and alpha bands, there exists more obvious asymmetry in the interbrain networks. In support of these findings, theta band coherence has been found to correlate with leader–follower relationships in cooperation [54]. It was also reported that differential roles could affect participants’ interbrain networks greatly in the alpha band. As the parietal lobe integrates sensory information of tactile and visual modalities [60], the stronger functional connectivity in the parieto-occipital areas for leaders can be considered as multimodal integration of information. Moreover, the increased involvement of left central regions represents the activation of somatosensory regions [61,62]. Notably, compared with the coupling patterns in the 2 single-factor contact conditions, interbrain coupling patterns in the eye and hand contact condition are stronger and show the trend of concentration toward left prefrontal areas and left frontocentral areas obviously. Since that, it can be assumed that left prefrontal areas involved cognitive control and left frontocentral areas involved behavior prediction during the mutual eye contact and right hand contact. In support of the assumption, it was found that there appeared increased interpersonal coherence in the superior frontal cortex during cooperation [63], and the frontal cortex also played an important role in empathy and theory of mind [64]. This study provides us with a new perspective to explore the variance of nonphysical and physical interaction.

The hypothesis behind improving BCI decoding accuracy by using additional sensory input is that the perception of multineural information from tactile and visual feedback can greatly enhance neural activities, such as EEG power changes (Fig. 2) and ERD improvement (Fig. 3) in this study. BCI decoding results in this study also prove that social interaction contributes positively to BCI performance compared with no-contact condition. Notably, the significant increase in decoding accuracy was only observed in eye contact condition and eye and hand contact condition but absent from hand contact condition. As shown in Fig. 2, significant power changes were found during eye contact condition compared with no-contact condition. Besides, in the eye and hand contact condition, the involved cortical areas were similar to those in the eye contact condition, which indicated that eye contact plays a relatively more important role. ERD reflects a decrease of oscillatory activity related to internally or externally paced events, and only channels within the region of interests were included for statistical analysis, such as significant ERD changes for C3 in the hand contact condition and eye and hand contact condition (Fig. 3). Both the global EEG power changes and ERD contributed to the decoding accuracy improvement.

Clarifying the mechanisms of social interaction on BCI training is the first step toward a broader and more effective applications in social settings. For example, medical treatments typically occur in the framework of a social interaction between health care providers and patients. Beneficial clinical interactions may enhance BCI performance by incorporating brain-to-brain coupling, thus maximizing treatment effects of BCI therapy in clinical applications, such as stroke rehabilitation. A limitation of this study is that the proposed method was only tested in a healthy population and has not been applied in clinical applications. The sample size was also comparatively small. Therefore, future large-scale trials should be considered and the potential of the proposed clinical use should be verified.

This study explored the contributions of social interaction to MI-based BCI training. By comparing EEG power, ERD, interbrain coupling connectivity, and BCI performance, we have provided evidence that social interaction can facilitate cortical activities, enhance brain activation and promote interbrain coupling. Adding social interaction in BCI-based robot control may further promote BCI performance in conjunction with neural synchronization between users.

## Data Availability

All custom codes as well as EEG data are available with a formal data sharing agreement.
