# Supplementary material for: Enhancing Brain–Computer Interface Performance by Incorporating Brain-to-Brain Coupling
Source: Cyborg Bionic Syst. 2024 Apr 25;5:0116. doi: 10.34133/cbsystems.0116 (PMC11052607; doi:10.34133/cbsystems.0116)
Supplement: Supplementary 1 — Figs. S1 to S4 [file cbsystems.0116.f1.pdf]

Supplementary Materials for

Enhancing brain-computer interface performance by incorporating brain-to-brain coupling

Tianyu Jia†, Jingyao Sun†, Ciarán McGeady, Linhong Ji, Chong Li*

Corresponding author: chongli@tsinghua.edu.cn

The file includes:

Fig. S1. Control group: the power for each electrode under the four experimental conditions in the alpha frequency band.
Fig. S2. Control group: comparisons of the grand average temporal ERD (C3) under the four conditions.
Fig. S3. Control group: comparisons of the grand average mean ERD (C3) under the four conditions.
Fig. S4. Control group: comparisons of BCI decoding accuracies under the four conditions.

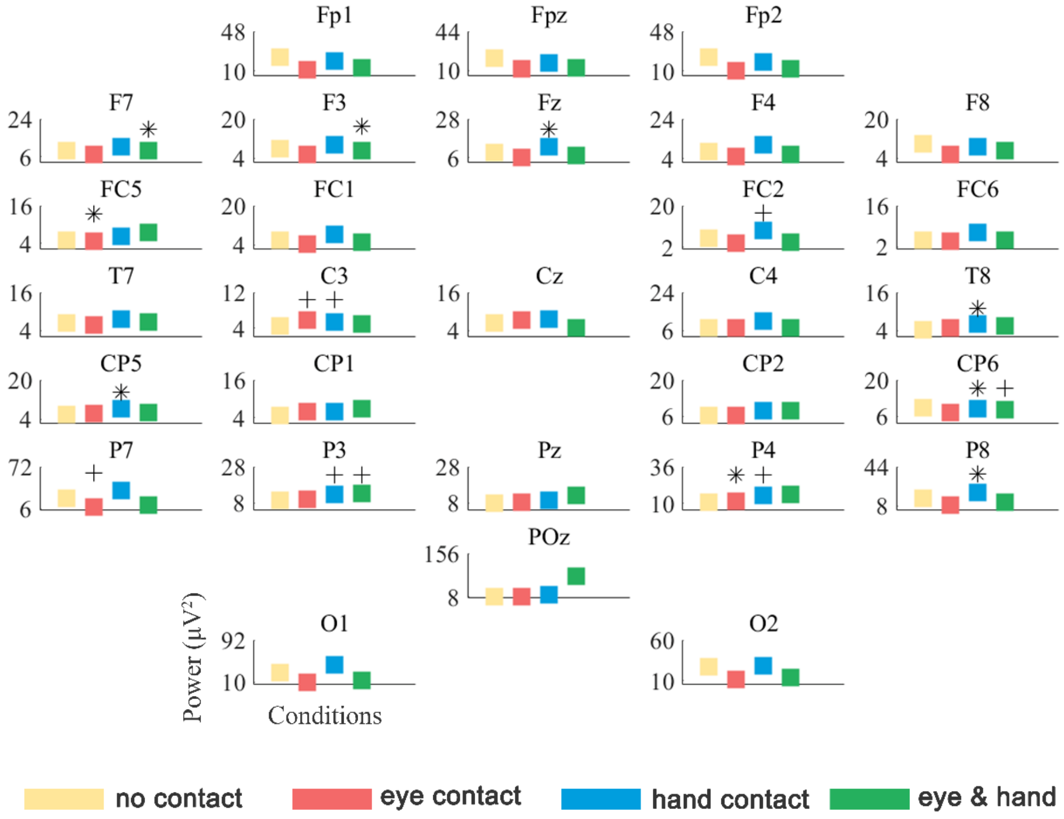

Fig. S1. Control group: the power for each electrode under the four experimental conditions in the alpha frequency band. ∗ denotes a significant difference between the session with contact and the session without contact ($p<0.05$), + denotes a difference that tended to be significant between the session with contact and the session without contact ($p<0.1$)

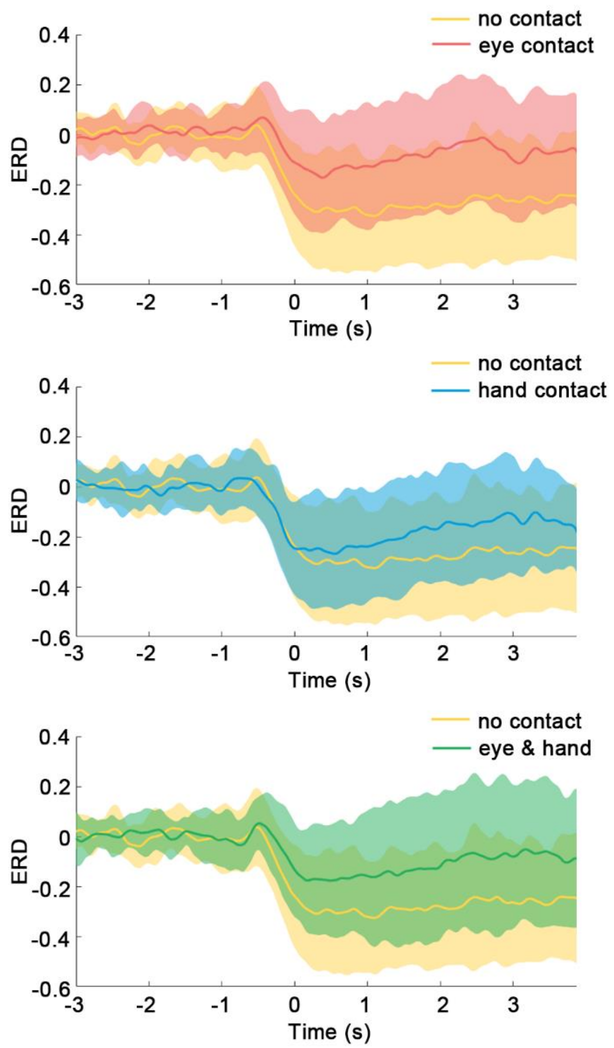

Fig. S2. Control group: comparisons of the grand average temporal ERD (C3) under the four conditions. The shaded region represents standard deviation of temporal ERD across subjects and the solid line represents the average value.

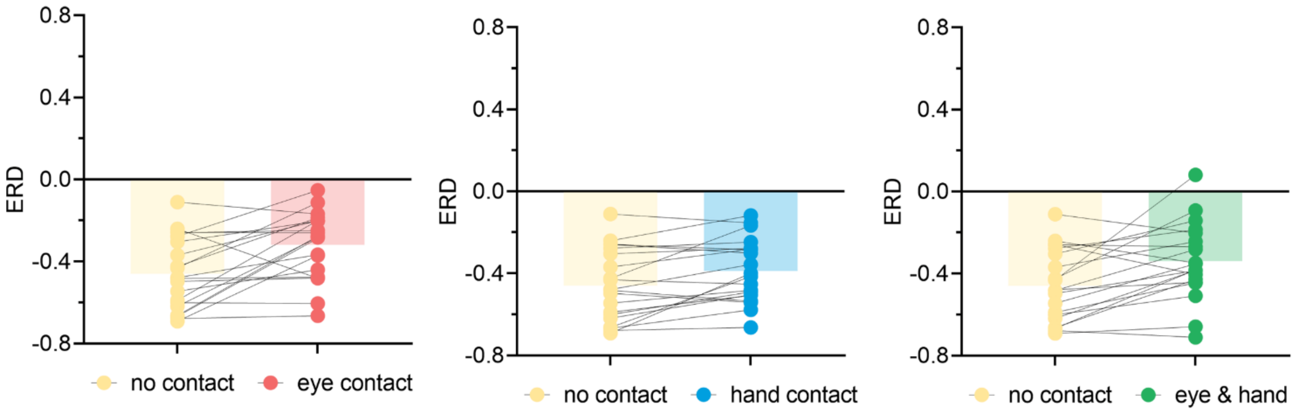

Fig. S3. Control group: comparisons of the grand average mean ERD (C3) under the four conditions.

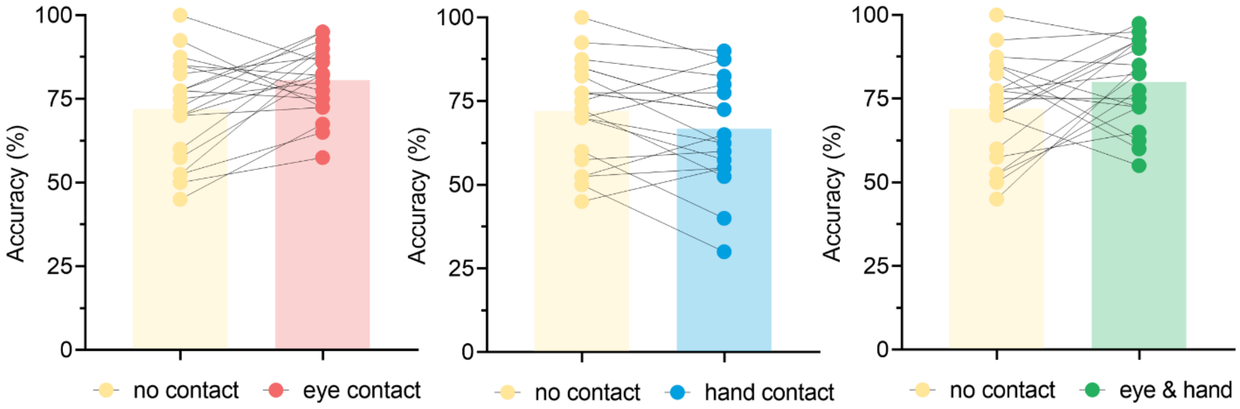

Fig. S4. Control group: comparisons of BCI decoding accuracies under the four conditions.
